# A role for tunneling nanotubes in virus spread

**DOI:** 10.3389/fmicb.2024.1356415

**Published:** 2024-02-16

**Authors:** Weimiao Lv, Zichen Li, Shule Wang, Jingyi He, Leiliang Zhang

**Affiliations:** ^1^Department of Clinical Laboratory Medicine, The First Affiliated Hospital of Shandong First Medical University and Shandong Provincial Qianfoshan Hospital, Jinan, Shandong, China; ^2^Department of Pathogen Biology, School of Clinical and Basic Medical Sciences, Shandong First Medical University and Shandong Academy of Medical Sciences, Jinan, Shandong, China; ^3^Medical Science and Technology Innovation Center, Shandong First Medical University and Shandong Academy of Medical Sciences, Jinan, Shandong, China; ^4^School of Clinical Medicine, Shandong Second Medical University, Weifang, Shandong, China

**Keywords:** TNTs, actin, virus spread, SARS-CoV-2, HIV

## Abstract

Tunneling nanotubes (TNTs) are actin-rich intercellular conduits that mediate distant cell-to-cell communication and enable the transfer of various cargos, including proteins, organelles, and virions. They play vital roles in both physiological and pathological processes. In this review, we focus on TNTs in different types of viruses, including retroviruses such as HIV, HTLV, influenza A, herpesvirus, paramyxovirus, alphavirus and SARS-CoV-2. We summarize the viral proteins responsible for inducing TNT formation and explore how these virus-induced TNTs facilitate intercellular communication, thereby promoting viral spread. Furthermore, we highlight other virus infections that can induce TNT-like structures, facilitating the dissemination of viruses. Moreover, TNTs promote intercellular spread of certain viruses even in the presence of neutralizing antibodies and antiviral drugs, posing significant challenges in combating viral infections. Understanding the mechanisms underlying viral spread via TNTs provides valuable insights into potential drug targets and contributes to the development of effective therapies for viral infections.

## 1 Introduction

The tunneling nanotube (TNT) was first proposed in 2004 as a unique pathway for direct cell-to-cell interaction (Rustom et al., 2004). It was described as a structure abundant in F-actin, with diameters ranging from 50 to 200 nm. Actin-driven cellular protrusions were suggested to reach out to neighboring cells and participate in TNT formation. These connections were found to be highly sensitive to light exposure, mechanical stress, and chemical fixation (Rustom et al., 2004). Similar communication channels, known as cytonemes, were observed in the Drosophila wing imaginal disc (Ramírez-Weber and Kornberg, 1999). Notably, TNTs were also discovered in different cell types, such as human endothelial progenitor cells (Koyanagi et al., 2005), neuronal cells (Sun et al., 2012), and immune cells (Watkins and Salter, 2005), indicating their diverse functions. Subsequent studies confirmed that TNTs could transport various cargos, including calcium signals (Watkins and Salter, 2005), proteins (Sisakhtnezhad and Khosravi, 2015), MHC class I molecules (Schiller et al., 2013b), organelles (Bukoreshtliev et al., 2009), and even viruses (Sowinski et al., 2008). It is worth noting that the concept of tunneling nanotubes (TNTs) was first proposed in a 2004 paper (Rustom et al., 2004). Since then, it has been observed that various neurodegenerative diseases, including Parkinson’s, Huntington’s, and Alzheimer’s diseases, can be spread through the transport of faulty proteins via TNTs (Abounit et al., 2016a,b; Tiwari et al., 2021). Overall, TNTs play critical roles in normal cell function, cell communication, viral infection, and the development of various diseases (Lou et al., 2012; Abounit et al., 2016a).

**Figure 1:**
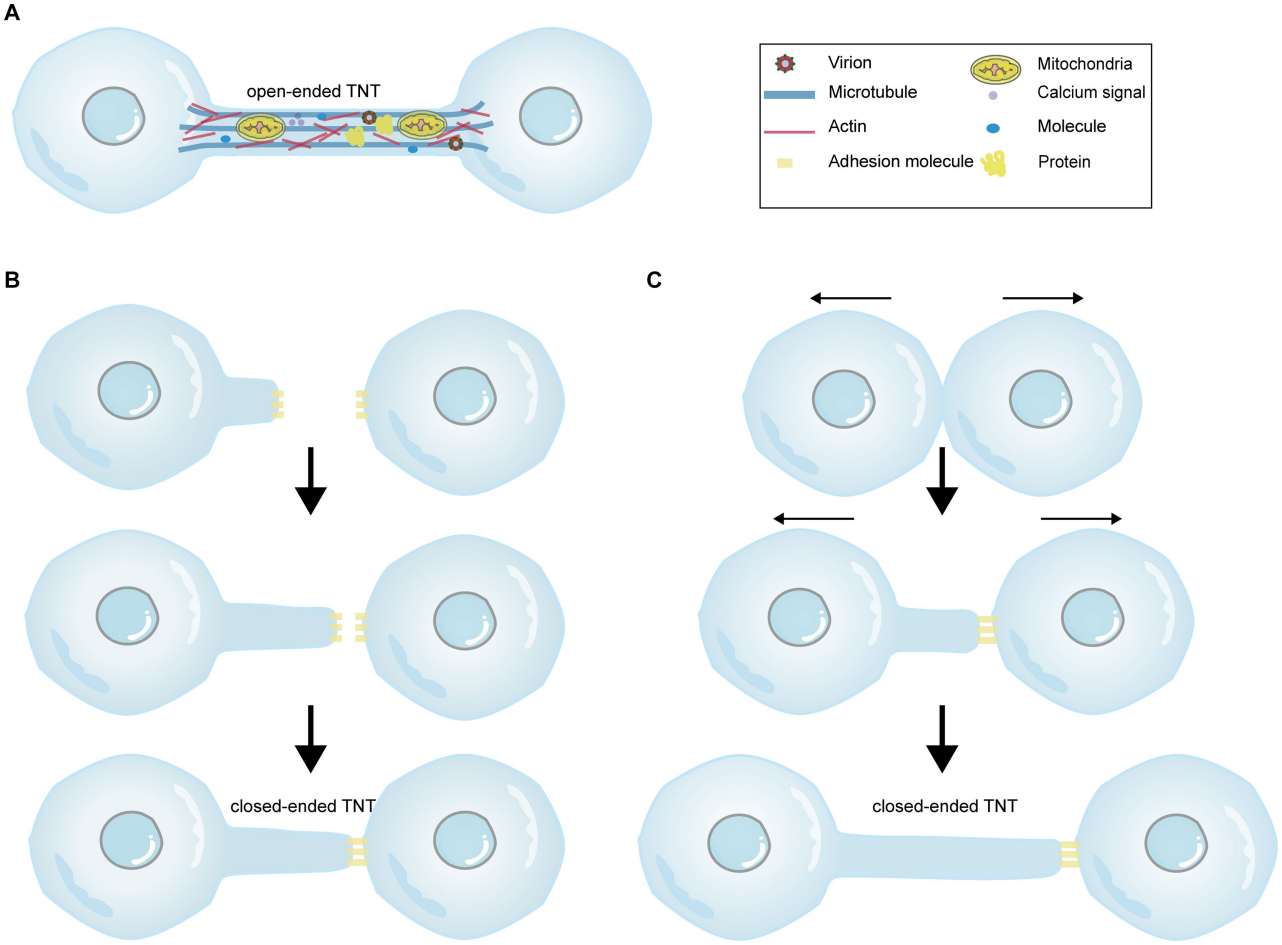
The diagram illustrating the cargos carried within TNTs and the mechanisms of TNT formation. There are two forms of TNTs, namely open-ended TNTs **(A)**, which allow cytoplasmic continuity between contacted cells, and closed-ended TNTs **(B,C)**. **(A)** Open-ended TNTs facilitate the transfer of various cargos between cells, including calcium signals, proteins, molecules, numerous organelles, and even viruses. **(B,C)** Depict two distinct mechanisms of closed-ended TNT formation. **(B)** The former demonstrates that actin-driven protrusions fuse with the membrane of another cell, forming closed-ended TNTs through adhesion molecules between cells. **(C)** The latter shows that contacted cells separate from each other and extend nanotubes. The Figure is generated through Adobe illustrator 2023.

Initially, it was believed that TNTs exclusively consisted of a single type of cytoskeletal component, F-actin (Rustom et al., 2004). However, subsequent studies have revealed that thicker TNTs also contain microtubules, with a diameter reaching 0.7 μm, in contrast to those that solely consist of F-actin (Onfelt et al., 2006). Microtubules are deemed to involve in cargo transport and provide TNTs with rigidity and longer lifespan (Austefjord et al., 2014). Moreover, myosin motors, including myosin Va and myosin X, are found within TNTs. Myosin Va facilitates the transport of organelles through TNTs (Rustom et al., 2004), while myosin X plays a role in TNTs formation (Gousset et al., 2013). Furthermore, TNTs can either be open (Figure 1A) or closed terminated (Figures 1B,C; Rustom et al., 2004; Sowinski et al., 2008). Four criteria have been proposed to identify TNTs (McCoy-Simandle et al., 2016; Jansens et al., 2017): (1) TNTs form a bridge between cells rather than being attached to the substratum like filopodia; (2) TNTs form a straight connection between cells; (3) TNTs contain F-actin; and (4) TNTs allow direct cell-to-cell communication through the transport of molecules or organelles. Additionally, nanotubes lack a midbody when observed by transmission imaging, distinguishing them from cytoplasmic bridges formed by cell division or filopodia (Sowinski et al., 2011). In general, TNT formation is believed to occur through two mechanisms: actin-driven protrusion and cell-dislodgment mechanism (McCoy-Simandle et al., 2016). The first proposed mechanism suggests that an actin-driven protrusion merges with the membrane or protrusion of another cell (Figure 1B; Rustom et al., 2004). The second viewpoint suggests that cells initially make contact and then separate from each other, extending nanotubes in the process (Figure 1C; Davis and Sowinski, 2008). Interestingly, these two processes can occur simultaneously without any conflict.

The molecular pathways governing TNT formation are complex, possibly attributed to their heterogeneous nature. For instances, M-Sec, also known as tumor necrosis factor-α-induced protein, is an important regulator involved in TNT formation between macrophages. Knockdown of M-Sec using short hairpin RNA (shRNA) reduces the number of TNTs and calcium flux through nanotubes. M-Sec promotes TNT formation by interacting with the small GTPase RalA and the exocyst complex (Hase et al., 2009). The N-terminal polybasic region of M-Sec localizes to the plasma membrane by binding to phosphatidylinositol (4,5)-bisphosphate. Subsequently, the positively charged surface of domains D and E at the C-terminus of M-Sec interacts with RalA, leading to the extension of the plasma membrane (Kimura et al., 2016). Additionally, leukocyte-specific transcript 1 (LST1) has been found to facilitate the interaction between RalA and the exocyst complex. LST1 also interacts with M-Sec, myosin, and myoferlin to induce the formation of nanotubes (Schiller et al., 2013a). Rho GTPases CDC42 and Rac1 contribute to the elongation process of TNT biogenesis, while actin nucleation factors actin related protein 2/3 (Arp2/3) and Wiskott-Aldrich syndrome protein (WASP) family verprolin-homologous 2 (WAVE2) play important roles in actin polymerization and TNT formation (Hanna et al., 2017). Interestingly, the interplay between oxidative stress and TNTs may play a pivotal role. A previous study has demonstrated that reactive oxygen species (ROS) can induce cytoskeletal reorganization and the formation of TNT-like communication structures in astrocytes by activating p38 MAPK (Zhu et al., 2005).

Besides their physiological functions in signal transduction, immune responses, embryogenesis, and cellular differentiation (Sisakhtnezhad and Khosravi, 2015), TNT-mediated intercellular communication has been implicated in various pathologies, including cancer (Rustom et al., 2004; Lou et al., 2012), neurodegenerative diseases (Abounit et al., 2016a,b), and pathogen transfer (Ganti et al., 2021; Jahnke et al., 2022; Pepe et al., 2022; Djurkovic et al., 2023; Yin et al., 2023). Increasing evidence suggests that TNT formation contributes to cell-to-cell transmission of viruses and facilitates rapid infection expansion compared to receptor-mediated fusion alone. In this review, we focus on the role of TNTs in the spread of various viruses (Table 1), such as retroviruses, Influenza virus, paramyxovirus, SARS-CoV-2, herpesviruses, and alphaviruses. TNT-mediated intercellular communications provide a significant opportunity for viruses to evade recognition from the immune system and neutralizing antibody activity. Additionally, we discuss viral and host proteins involved in TNT formation and the potential implications for antiviral strategies.

**Table 1:** Overview of TNTs or TNT-like structures induced by different viruses.

Virus	F-actin	Microtubule	Cell type	Viral proteins involved in TNT formation	Host proteins involved in TNT formtion	Potential inhibitors blocking virus spread	References
HIV-1	Yes	Yes	Macrophages, MDMs, T, B, DCs	Nef	Vav, Rho GTPase, PAK2, M-Sec, ERP29, Siglec-1, Cx43	ND	Xu et al. (2009), Imle et al. (2015), Okafo et al. (2017), and Pergu et al. (2019)
HTLV-1	Yes	No	MT-2, Jurkat, THP-1	p8	ND	Cytarabine	Omsland et al. (2018)
IAV	Yes	ND	A549, MDCK, Vero	ND	Rab11a	IPA-3, Cytochalasin D	Roberts et al. (2015) and Kumar et al. (2017)
HMPV	Yes	Yes	BEAS-2B, A549, Vero, 16HBE	P	Rho GTPases	ND	El Najjar et al. (2016)
NDV	Yes	Yes	DF-1, BSR-T7/5, Vero	F	Methyltransferase	ND	Li et al. (2023)
MV	Yes	ND	GCCM	ND	ND	ND	Duprex et al. (1999)
PIV5	Yes	ND	A549, MDCK, Vero	ND	ND	ND	Roberts et al. (2015)
SARS-CoV-2	Yes	Yes	Vero E6, SH-SY5Y	ND	CK2, p38 MAPK	ND	Pepe et al. (2022)
PRV	Yes	Yes	ST, RK13	US3	Rho GTPases, PAK	ND	Van den Broeke et al. (2009), Jacob et al. (2015), and Jansens et al. (2017)
HSV-1	Yes	Yes	HCECs, Vero	ND	ND	CK666	Wang et al. (2023)
BoHV-1	Yes	Yes	MDBK, KOP, bovine fibroblasts	US3	ND	ND	Panasiuk et al. (2018)
MHV-68	Yes	No	NIH-3 T3, BHK-21, 293 T cells	Gp48	ND	ND	Gill et al. (2008)
EBV	Yes	No	293 T, Cos7, HeLa	BMRF2, BDLF2	ND	ND	Loesing et al. (2009)
CHIKV, SINV, SFV	Yes	Yes	Vero, HUVEC, MEF	E2, capsid	ND	Bafilomycin A1, tetherin, Rab5DN	Martinez and Kielian (2016) and Yin et al. (2023)
PRRSV	Yes	Yes	HEK-293T, MARC-145	ND	ND	Cytochalasin D, ML7	Guo et al. (2016)
VACV	Yes	ND	Vero	F11L	ND	ND	Morales et al. (2008)
EBOV	Yes	Yes	Vero, HUVECs, Macrophages	NP, VP35, VP24	M-Sec	Cytochalasin D, nocodazole	Djurkovic et al. (2023)

ND, not determined.

## 2 The role of TNTs in viral spread

### 2.1 Retrovirus

Human immunodeficiency virus (HIV), especially HIV-1, is the causative agent of acquired immune deficiency syndrome (AIDS), which attacks CD4+ T cells and damages the immune system. Human T cells have been observed to come into contact with each other, separate, and form membrane nanotubes that are not attached to the substratum (Sowinski et al., 2008). This discovery provides a novel and rapid route for the spread of HIV-1. Interestingly, blocking the CD4 receptor or the gp120 subunit of the envelope protein does not impede the formation of nanotubes. However, the transfer of HIV to uninfected T cells is significantly reduced, indicating that T-cell nanotubes do not exhibit seamless flow; instead, they are closed-ended structures rather than open-ended tunnels (Sowinski et al., 2008). Unlike T-cell nanotubes without microtubules, subsequent studies revealed that macrophage TNTs contain microtubules (Eugenin et al., 2009), implying that TNTs may possess different structures and functions in distinct cell types. Interestingly, HIV infection was found to promote TNT formation in human macrophages, (Eugenin et al., 2009) distinguishing it from T cells. This mechanism is discussed below. Long TNTs facilitate the movement of the virus between macrophages. The virus undergoes rapid actin- and myosin-mediated transport, resembling a form of “surfing,” as the viral particle appears to be larger than the diameter of the long TNT (Eugenin et al., 2009).

**Figure 2:**
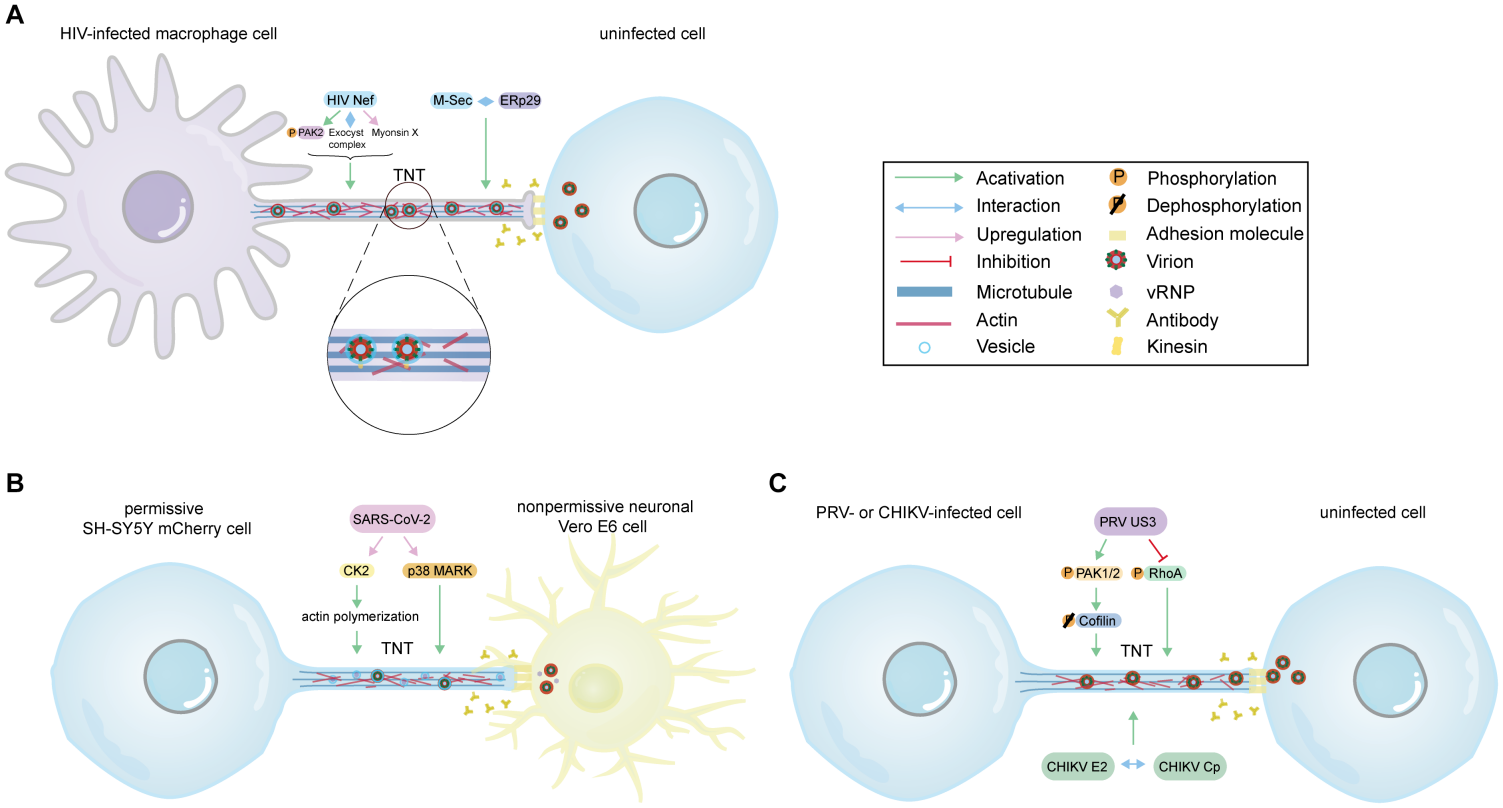
The model of virus-induced TNTs formation. **(A)** The model of HIV-induced TNTs in macrophages. Despite the presence of antibodies, HIV Nef induces TNTs formation by activating and phosphorylating PAK2, interacting with the exocyst complex, and upregulating myosin X expression levels. Additionally, M-Sec contributes to TNT formation by interacting with the ER chaperone ERp29. Virus particles, enveloped within vesicles, are transported along microtubules via kinesin and transferred to uninfected cells through TNTs. **(B)** The model of SARS-CoV-2-induced TNTs. SARS-CoV-2 infection increases the number of TNTs despite the presence of neutralizing antibodies. SARS-CoV-2 upregulates CK2, a positive factor for actin polymerization, and activates p38 MAPK to induce TNTs formation. Vesicular structures within TNTs may contain vRNP and mature virus, which can be transferred from SH-SY5Y mCherry cell to human nonpermissive neuronal Vero E6 cells via TNTs. **(C)** The model of TNTs induced by PRV (ST, RK13 cells) or CHIKV (Vero, HUVEC, MEF cells). PRV US3 participates in cytoskeletal rearrangement and virus propagation by phosphorylating and activating CDC42/Rac1 signaling downstream effectors PAK1/2, and subsequently dephosphorylating Cofilin. Moreover, US3 phosphorylates RhoA signaling to stimulate the formation of TNTs. The interaction between the E2 protein and the capsid protein is essential for the formation of TNTs during CHIKV infection. **(A–C)** Provide evidence supporting the notion that TNTs serve as an effective pathway for the virus to evade the immune system’s defense mechanisms, including antibody neutralization. The Figure is generated through Adobe illustrator 2023.

The formation of TNTs in HIV-infected macrophages relies on the HIV immunosuppressive protein Nef and the cellular protein M-Sec. Nef induces actin remodeling by interacting with the guanine nucleotide-exchange factor Vav in a Vav-mediated GTPase-dependent pathway (Xu et al., 2009). Furthermore, depletion of specific members of the exocyst complex impairs Nef-induced TNT formation, highlighting the significance of the exocyst complex (Mukerji et al., 2012). Subsequent studies suggest that Nef contributes to TNT formation by activating p21-activated kinase 2 (PAK2), which regulates Aurora-A to phosphorylate RalA (Figure 2A; Imle et al., 2015). Another study also indicates that Nef enhances TNTs in a Myo10-dependent manner in macrophages or human monocyte-derived macrophages (Figure 2A; Uhl et al., 2019). Additionally, the cellular protein M-Sec has been identified as a positive regulator of Nef-mediated HIV-1 transmission via TNTs (Lotfi et al., 2020). M-Sec participates in TNT formation by interacting with the necessary endoplasmic reticulum (ER) chaperone ERp29 (Figure 2A; Hashimoto et al., 2016; Pergu et al., 2019). Moreover, Nef plays a significant role in AIDS progression and immune dysfunction. It has been reported that Nef can be transferred from infected macrophages to B cells through TNTs, thereby inhibiting class switching (Xu et al., 2009). This confirms that TNTs serve as a novel mechanism for HIV to disrupt the antiviral IgG and IgA response, evading humoral immunity. Taken together, these findings imply that Nef and M-Sec play an essential role in TNT formation and HIV transmission. Although inhibiting M-Sec decreases Nef-mediated TNT formation, the mechanisms by which Nef acts as an upstream molecule and regulates M-Sec are not yet well understood.

Interestingly, in the context of tuberculosis (TB), which is the most common co-infection with HIV-1, the TB microenvironment has been found to exacerbate the spread of HIV-1 (Souriant et al., 2019). These findings suggest that TB promotes the increase of M2 macrophages with IL-10 [M(IL-10)], regulates IL-10/STAT3 signaling, enhances TNT formation, and thereby increases intercellular communication to facilitate HIV-1 infection. Siglec-1 is also involved in TNTs-mediated virus infection. Compared with thin TNTs, Siglec-1 mainly localizes on microtubule-containing thick TNTs with more viral particles (Dupont et al., 2020). In the tuberculosis context, high amounts of type I interferon (IFN-I) increases Siglec-1 expression, induces exacerbation of HIV-1 infection, and spread in macrophages (Dupont et al., 2020). A previous study proposed gap junctions (GJs), which were required for the formation of the synaptic contact, as a critical mediator of HIV infectivity via TNTs (Eugenin et al., 2009). And connexin-43 (Cx43) localized at the base and tip of the TNTs. Notably, Cx43 localized at the tip of the TNTs mediates functional GJ communication in the context of HIV. Blocking TNT or GJ communication significantly diminishes HIV-p24 expressing cells, suggesting that TNTs and GJs are required for HIV propagation (Okafo et al., 2017). Exploring strategies to target GJs and TNTs could represent a promising approach to mitigate the spread of HIV. Despite the effectiveness of antiretroviral therapy, HIV can still propagate through TNTs within tissues (Okafo et al., 2020). Therefore, gaining a deeper understanding of the mechanisms underlying HIV transmission via TNTs may not only improve the efficacy of current antiretroviral therapies but also offer new possibilities for the treatment of AIDS.

In addition to T lymphocytes and macrophages, CD40L associated with Th cells can also stimulate dendritic cells (DCs) to form unique branching TNTs. While these TNTs facilitate intercellular communication among immune cells, they can also be exploited by HIV for spreading infection (Zaccard et al., 2015). It is well known that complement-opsonized HIV (HIV-C) can infect DCs by evading SAMHD1, which depletes the virus (Posch et al., 2015). Interestingly, a study has described the involvement of complement in TNT formation (Bertacchi et al., 2022). HIV-C enhances the formation of TNTs in both DCs and DC/CD4+ T-cell co-cultures, significantly increasing their quantity compared to HIV alone. Local generation of C5a and C3a occurs upon HIV infection. C5a receptor stimulates TNT formation and HIV transfer, whereas C3a receptor does not exert any effect (Bertacchi et al., 2022). This finding suggests that C5a receptor could be a potential target for developing treatment strategies against HIV.

Human T-cell leukemia virus type 1 (HTLV-1) is an oncogenic retrovirus that can lead to prolonged carrier status and cancer in humans. CD4+ memory T cells are the primary cellular targets of HTLV-1. Cell-to-cell interactions are considered the main and most efficient mode of HTLV-1 transmission and dissemination. HTLV-1 protein p8 could enhance intercellular conduits and facilitate viral cell-to-cell transmission (Malbec et al., 2011). Further studies show that HTLV-1 protein p8 increases viral transmission between T cells through TNTs containing viral proteins Tax and Gag. It should be noted that the use of cytarabine as a treatment option has been proposed to reduce the quantity of TNTs and subsequently decrease the spread of the virus, while not affecting p8 expression (Omsland et al., 2018). To summarize, TNTs can shield HTLV-1 from the immune system, while cytarabine has been shown to inhibit TNTs-mediated cell-to-cell transmission.

### 2.2 Influenza virus

Influenza A virus (IAV), which belongs to the *Orthomyxoviridae* family, is an enveloped virus with a negative-strand segmented RNA genome. It causes human influenza, a respiratory disease. Using a confocal microscope, researchers have observed that IAV-infected cells form intercellular connections containing F-actin and various IAV proteins, including IAV Hemagglutinin (HA), Nucleoprotein (NP), Matrix protein (M), Matrix protein 2 (M2), and viral ribonucleoprotein (vRNP) (Roberts et al., 2015). These connections serve as the minimal replication machinery, suggesting the existence of TNTs in IAV infection. Furthermore, the researchers have discovered that the influenza virus genome is also present in TNTs between infected and uninfected cells. By using IPA-3, a compound that inhibits PAK1 and affects actin dynamics, the number of these intercellular connections decreases, indicating that their formation relies on actin dynamics (Roberts et al., 2015). To investigate the role of tubulin, further experiments were conducted. Treatment with microtubule stabilizer paclitaxel reduces the formation of intercellular connections, demonstrating the involvement of the microtubule cytoskeletal network. Moreover, confocal microscopy has revealed that IAV-infected cells possess fewer microtubules compared to mock-infected cells (Roberts et al., 2015). These findings suggest that microtubules undergo depolymerization during IAV infection. Interestingly, a separate study conducted in 2017 showed that cellular extensions in epithelial cell lines (A549 and MDCK) contain tubulin, as indicated by labeling TNTs with alpha-tubulin (Kumar et al., 2017). These results indicated that different cell types, growth conditions, experimental methods, and timeframes may influence the presence of tubulin within TNTs. Blocking viral cell-free spread with hemagglutination-inhibiting antibodies and the neuraminidase enzyme inhibitor Oseltamivir has demonstrated that the influenza virus genome and proteins can transfer and spread through TNTs (Kumar et al., 2017). This direct transport suggests evasion strategies employed by IAV. The precise mechanism of IAV spread via TNTs remains of great interest. Previous research has shown that Rab11a, a small GTPase, plays a crucial role in the cytoplasmic transport of IAV viral RNA (Amorim et al., 2011). Additionally, Rab11a has been found to mediate bidirectional vRNP transport through TNTs in the context of IAV infection, leading to cellular coinfection and reassortment (Ganti et al., 2021). TNTs and the presence of Rab11a are essential for the transfer of IAV genomes. However, it is important to note that Rab11a alone does not mediate the generation of TNTs during IAV infection. Nonetheless, the search for potential drugs targeting Rab11 could be significant in preventing IAV transport within the cytoplasm and transmission to uninfected cells.

### 2.3 Paramyxovirus

Human metapneumovirus (HMPV), a member of *Paramyxoviridae*, is a human respiratory pathogen that causes severe respiratory disease. HMPV infection leads to the formation of branched filamentous networks and intercellular extensions through remodeling of the cell cytoskeleton. This process requires active actin dynamics and signaling involving Rho GTPases, including CDC42, Rac1, and RhoA. Additionally, the HMPV P protein co-localizes with actin and induces membrane extensions. Through co-culture assays, researchers have confirmed that HMPV can spread directly from cell to cell via actin-based intercellular extensions in a manner independent of neutralizing antibodies (El Najjar et al., 2016), which suggests the possibility of TNTs’ existence.

Newcastle disease virus (NDV) is a member of the *Paramyxoviridae* family, causing Newcastle disease and posing a severe threat to the poultry industry. By blocking alternative transmission routes, researchers have discovered that the NDV NP protein is located within TNTs that consist of both F-actin and tubulin, confirming the ability of NDV to propagate through TNTs (Li et al., 2023). As expected, viral proteins, particularly the F protein, are responsible for promoting the formation of TNTs. In addition, it has been noted that mutations in the methyltransferase K-D-K-E motif can prevent direct cell-to-cell transmission of NDV via TNTs (Li et al., 2023). Interestingly, methyltransferase motifs have also been identified in coronaviruses and Ebolavirus, offering valuable insights into virus dissemination of those two viruses (Jin et al., 2013; Valle et al., 2020).

Measles virus (MV) belongs to the *Paramyxoviridae* family and has a single-stranded RNA genome. Researchers have observed that glial cells infected with MV have the ability to fuse with uninfected cells through connecting processes that resemble TNT structures. This mechanism allows for cell-to-cell infection without the requirement of virus budding (Duprex et al., 1999). This mode of transmission enhances the chances of viral spread, emphasizing the need for further studies to validate the existence of TNTs.

Parainfluenza Virus 5 (PIV5), which belongs to the *Paramyxoviridae* family, is a prevalent respiratory pathogen capable of causing respiratory tract infections in various animals, including humans, cattle, and pigs. During PIV5 infection, there is a notable increase in the abundance of TNTs. Even in the presence of neutralizing antibodies, PIV5 effectively utilizes intercellular connections to propagate between cells (Roberts et al., 2015). TNTs enable the virus to bypass certain steps and evade immune surveillance, underscoring the broader role of TNTs in facilitating the swift dissemination of Paramyxoviruses. Elucidating the mechanism by which Paramyxoviruses infect cells through TNTs and their immune evasion strategies opens up new possibilities for drug development.

### 2.4 SARS-CoV-2

Severe acute respiratory syndrome coronavirus 2 (SARS-CoV-2), which belongs to the Betacoronavirus genus, group 2, is the pathogen responsible for the COVID-19 pandemic (Hu et al., 2021). Several studies have shown that SARS-CoV-2 can cause fever, cough (Chen N. et al., 2020), and some neurological complications by invading the central nervous system (CNS) (Hosseini et al., 2021). However, it is not clear how SARS-CoV-2 spreads in the human brain. In fact, ACE2 receptor levels are very low in most areas of the human brain (Chen R. et al., 2020), and the virus cannot enter neuronal cells through the receptor-mediated endocytosis pathway. It is worth investigating whether there are new mechanisms of virus invasion. Previous researches have indicated that the actin cytoskeleton, including actin filaments (AFs), microtubules (MTs), and intermediate filaments (IFs) (Wen et al., 2020), plays a role in the fusogenic activities of some coronaviruses, such as transmissible gastroenteritis virus (TGEV) and neurotropic porcine hemagglutinating encephalomyelitis virus (PHEV) (Hu et al., 2016; Lv et al., 2019; Zhang et al., 2020). This may play a role in the entry, replication, and maturation processes of SARS-CoV-2. Furthermore, a study using high-resolution scanning electron microscopy revealed that SARS-CoV-2 viral particles were wrapped with thin cellular projections resembling nanotubes (Caldas et al., 2020). In fact, like other viruses previously discussed, a study conducted in 2022 demonstrated that SARS-CoV-2 is capable of spreading between permissive infected cells and nonpermissive neuronal cells through a direct cell-to-cell contact-dependent pathway, specifically involving TNTs as depicted in Figure 2B (Pepe et al., 2022). SARS-CoV-2 can also utilize TNTs to propagate between permissive cells, bypassing the endocytic pathway and achieving higher transmission speed due to the prompt polymerization and depolymerization of actin in TNTs (Pepe et al., 2022). Researchers used cryo-correlative light and electron microscopy (CLEM), cryo-electron microscopy (cryo-EM), and cryo-electron tomography (cryo-ET) techniques to observe many vesicular structures inside TNTs, consisting of single and actin-rich tubes, which might carry replicative complexes and mature virus (Pepe et al., 2022). These findings suggest that TNTs serve as an effective strategy for SARS-CoV-2, enabling the virus to invade nonpermissive cells and enhance infection in permissive cells, thereby facilitating the broader dissemination of the virus. TNTs are also suggested to serve as a means of hiding the virus, functioning as an immune evasion strategy (Rubio-Casillas et al., 2022). Overall, based on the available information, it is evident that SARS-CoV-2 can exploit TNTs to contribute to central nervous system manifestations of COVID-19 and evade immune recognition. Previous studies have shown that Coronaviridae family viruses, such as TGEV and PHEV, promote F-actin polymerization through Rac1/Cdc42 GTPases (Zhang et al., 2020), which may be involved in SARS-CoV-2-mediated TNT formation. Additionally, SARS-CoV-2 can upregulate casein kinase II (CK2) and p38 MAPK activity to induce TNT formation (Zhu et al., 2005; Bouhaddou et al., 2020). CK2 may increase TNTs by promoting actin polymerization (Fernández-Golbano et al., 2014). Thus, drugs with inhibitory activity against Rac1/Cdc42 GTPases, CK2, and p38 MAPK could potentially impact virus propagation. Interestingly, a recent study discussed the similarities in molecular mechanisms and treatment approaches between cancer and COVID-19. They proposed that tumor-treating fields (TTFields) technology, an effective cancer treatment method, could also significantly inhibit TNT formation and weaken the ability of TNTs to promote SARS-CoV-2 entry and replication (Farmani et al., 2022). Consequently, targeting TNTs has emerged as a promising strategy to impede the spread of SARS-CoV-2.

### 2.5 Herpesviruses

*Alphaherpesviridae*, *Betaherpesviridae*, and *Gammaherpesviridae* are three subfamilies of herpesviruses, which are DNA viruses with an envelope and double-stranded genome. Increasing studies have highlighted the importance of US3 in TNT-mediated intercellular transmission and anti-apoptotic activity of alphaherpesviruses, such as pseudorabies virus (PRV), bovine herpesvirus 1 (BoHV-1), BoHV-5, Herpes simplex virus type 1 (HSV-1), HSV-2, and others. US3, a conserved viral serine/threonine kinase among the *Alphaherpesvirinae* subfamily, is associated with cytoskeletal rearrangement and enhanced spread (Favoreel et al., 2005). In a study conducted in 2017, a co-culture system and live-cell imaging techniques were employed to demonstrate that cells transfected with US3 were capable of forming projections and transmitting molecular information, even without the expression of other viral proteins (Jansens et al., 2017). In contrast, mutant cells lacking active US3 did not exhibit such capabilities. Through analysis using confocal microscopy, it was observed that US3-induced cell projections could form actin-containing cellular connections associated with TNTs (Jansens et al., 2017). Unlike the fragile characteristics of TNTs observed in earlier studies (Rustom et al., 2004), US3-induced stable TNTs were shown to be able to maintain for 24 h (Jansens et al., 2017). Immunostaining demonstrated that US3-induced TNTs contained post-translational modifications (PTMs), including acetylated tubulin and detyrosinated tubulin, which contributed to microtubule stabilization (Jansens et al., 2017). Additionally, transmission electron microscopy (TEM) observations have revealed the presence of adhesion molecules like E-cadherin and beta-catenin in the connection area of both pseudorabies virus (PRV)-infected cells and US3-transfected cells. These molecules may contribute to the stability of TNTs, ensuring persistent TNT-mediated contact (Jansens et al., 2017). It is well known that herpesviruses can be transmitted not only as cell-free particles but also directly from cell to cell. In the presence of neutralizing antibodies that block cell-free virion entry, experiments have demonstrated the dynamic movement of infectious BoHV-1 particles from infected cells to uninfected cells through these thin, long membrane protrusions, confirming the involvement of TNTs in BoHV-1 transmission (Panasiuk et al., 2018). The US3 protein of BoHV-5 also induces cytoskeletal reorganization, resulting in the formation of cell projections and interconnections resembling the structure of TNTs (Ladelfa et al., 2011). Recent studies have confirmed that the human-specific virus HSV-1 can spread through TNTs in addition to cell–cell spread and cell-free release. Upon infecting HCECs and Vero cells with wild-type HSV-1, the presence of TNTs was observed using F-actin-tracker to label the connections between cells at 24 h. The role of the Arp2/3 complex, known for its involvement in F-actin assembly, was investigated using the potent inhibitor CK666. The results demonstrated that CK666 significantly reduced the number of TNTs in two different cell types and weakened intercellular transmission of HSV-1 (Wang et al., 2023).

Regarding the specific mechanism of how US3 promotes the formation of TNTs, it has been found that PRV US3 possesses the ability to phosphorylate and activate PAK1/2, which are downstream effectors of CDC42/Rac1 signaling. This activation leads to the dephosphorylation of cofilin, ultimately resulting in TNT formation, as illustrated in Figure 2C (Van den Broeke et al., 2009; Jansens et al., 2020). On the other hand, US3 can phosphorylate and suppress PKA-dependent RhoA signaling, thereby promoting TNT formation (Figure 2C; Jacob et al., 2015). Moreover, transmission of PRV virions via TNTs was observed through TEM analysis, which revealed that the virions were packaged in transport vesicles (Figure 2C). This packaging process relies on the fusion envelope proteins gB (Jansens et al., 2017). Additionally, the presence of envelope glycoprotein D (gD), envelope glycoprotein E (gE), and viral particles in TNTs suggests their involvement in HSV-1 transmission (Wang et al., 2023). This indicates that virions may undergo exocytosis to establish contact with uninfected cells, and this process is likely applicable to the transportation of particles from other alphaherpesviruses as well. Additionally, Miro1, a mitochondrial Rho GTPase 1, has been proposed to be involved in the movement of mitochondria between nonneuronal cells via TNTs (Ahmad et al., 2014). PRV has the ability to disrupt mitochondrial dynamics by targeting Miro1, which could potentially hinder the rescue of apoptotic cells. Similarly, the expression of HSV-2 US3 leads to the reorganization of the actin cytoskeleton and the formation of filamentous processes, resembling the effects observed in PRV (Finnen et al., 2010).

It is interesting to note that autophagy, as an essential component of innate and adaptive immunity, has been shown to be significant in the anti-apoptotic activity of US3. There is evidence to suggest that US3 targets independent autophagy regulators and inhibits autophagy in HSV-1-infected epithelial cells and fibroblasts (Rubio and Mohr, 2019). PAK1 also plays a limited but important role in the anti-apoptotic activity of the PRV US3 protein kinase (Van den Broeke et al., 2011). Moreover, US3 can activate the AKT/mTOR pathways in PRV infected cells to reduce the level of autophagy (Sun et al., 2017). Focusing on US3 may provide novel insights into anti-virus strategies. In an early 2001 study, it was found that inserting lacZ or egfp at the alphaherpesvirus US4 gene-encoded glycoprotein G (gG) locus decreased expression of the upstream US3 protein and inhibited cell-to-cell virus spread (Demmin et al., 2001). BX795 has been shown to inhibit duck plague virus (DPV) US3 kinase activity, reduce the phosphorylation of US3 substrates, and impede TNTs formation (Tian et al., 2023).

Other members of the herpesvirus family include the *gammaherpesviruses* Epstein–Barr virus (EBV) and mouse gammaherpesvirus 68 (MHV-68). *Gammaherpesviruses* possess a conserved open reading frame 27 (ORF27) that encodes gp48, which promotes direct viral intercellular propagation (May et al., 2005). ORF58 stimulates gp48 transport, and gp48 is involved in actin cytoskeleton reorganization, but not microtubules, which requires Rho GTPase function. This eventually leads to the formation of membrane protrusions and promotes virus spread via TNTs (Gill et al., 2008). EBV glycoproteins BMRF2 and BDLF2 cause cytoplasmic protrusions that depend on the actin skeleton, lamellipodia, and increase virion dissemination between cells (Loesing et al., 2009). Therefore, it is hypothesized that herpesviruses employ TNTs as a rapid mode of cell-to-cell transmission.

### 2.6 Alphaviruses

Alphaviruses, which belong to the *Togaviridae* family, are small enveloped positive-strand RNA viruses, including chikungunya virus (CHIKV), sindbis virus (SINV), and Semliki Forest virus (SFV). Alphaviruses consist of a nucleocapsid core composed of the RNA genome surrounded by capsid protein (Cp), as well as a viral envelope that contains the E2 and E1 transmembrane proteins. Moreover, alphavirus infection leads to significant modifications in the host cell cytoskeleton, and through confocal microscopy, long-actin/tubulin positive intercellular extensions resembling flattened tips can be observed in Vero cells or primary human umbilical vein endothelial cells (HUVEC) (Martinez and Kielian, 2016). The formation of TNT-like structures was found to require the expression and interaction of E2 and Cp proteins (Figure 2C), suggesting that cell-to-cell transfer relies on the production of fusion-active virus particles rather than viral RNA or replication (Martinez and Kielian, 2016). It is important to note that the membrane between the infected cell and the recipient cell does not fuse, indicating that virus spread via TNTs may involve processes such as exocytosis and endocytosis, similar to intercellular transmission observed in alphaherpesviruses. One question that arises is how the interaction between E2 and Cp proteins mediates changes in the cytoskeletal network and subsequent formation of TNTs. The latest article has provided a definition of intercellular long extensions (ILE)/TNT observed in CHIKV-infected cells (Yin et al., 2023). Consistent with the findings of a study conducted in 2016 (Martinez and Kielian, 2016), it was observed that the extensions between infected and target cells do not fuse. Instead, they appear to form closed, terminated TNT-like structures. However, this particular article aimed to reduce viral intercellular transmission by blocking the endocytic pathway. Exploring potential drugs that can hinder dynamin-mediated vesicle scission, Rab5-mediated delivery to early endosomes, and exposure to endosomal low pH may effectively decrease CHIKV cell-to-cell propagation. Interestingly, CHIKV cell-to-cell transmission in mice also displayed resistance to antibody-mediated inhibition (Yin et al., 2023). Further research on the mechanisms underlying virus spread through intercellular extensions will contribute to the development of antiviral therapeutics.

### 2.7 Others

Porcine reproductive and respiratory syndrome virus (PRRSV), a member of the *Arteriviridae* family, is an enveloped positive-sense, single-stranded RNA virus. By employing confocal microscopy, researchers have discovered long intercellular nanotubes containing F-actin and myosin IIA between neighboring cells, and PRRSV infection promotes the formation of these nanotubes between infected and uninfected cells (Guo et al., 2016). Furthermore, various PRRSV proteins, including nsp1β, nsp2, nsp2TF, nsp4, nsp7, and nsp8, as well as replication and transcription complexes (RTC) and viral RNA, have been observed to be transported via TNTs. Interestingly, PRRSV has the ability to spread through intercellular nanotubes even when virus-neutralizing antibodies are present, bypassing the need for cell-free virions (Guo et al., 2016). This highlights the ability of TNTs to evade the immune system. Furthermore, transfection experiments have shown that the transfer of PRRSV through nanotubes does not rely on host cell receptors, which aligns with the findings of SARS-CoV-2 and CHIKV infections via TNTs mentioned earlier (Guo et al., 2016). Moreover, arterivirus GP5 proteins have been found to be associated with TNTs, raising the question of whether and how GP5 is involved in TNT formation and the promotion of virus dissemination. It is worth noting that the expression of ROS and S100A4 mRNA, which are known to play a role in cell survival pathways, is elevated in PRRSV-infected cells. Previous studies have reported that ROS can enhance the formation of nanotubes (Zhu et al., 2005; Leclerc et al., 2007). Additionally, it has been observed that mitochondria, which are crucial regulators of cell survival and cell death, can be transferred from uninfected cells to PRRSV-infected cells through TNTs (Guo et al., 2018). This phenomenon may provide opportunities for cell survival in the early stage of infection, allowing for ample viral replication in host cells. However, it is also possible that mitochondria could serve as carriers to transport viral components to uninfected cells via TNTs, thereby facilitating the spread of infection. This mechanism could potentially rescue virus-stressed cells and delay cell death.

Vaccinia virus (VACV), a member of the *Orthopoxvirus* genus of the *Poxviridae* family, possesses a large DNA genome. The VACV F11L has been shown to induce actin rearrangements, promoting cell detachment, migration, and contributing to VACV cell-to-cell propagation (Morales et al., 2008). Additionally, F11L has the ability to block RhoA signaling, which is necessary for VACV-mediated cell motility, as described in another study referenced (Valderrama et al., 2006). An interesting area to explore is whether VACV can stimulate the formation of TNTs. Immunofluorescence assays conducted in a separate research study have provided evidence suggesting that VACV-induced actin rearrangement may potentially lead to the formation of TNTs rich in F-actin (Xiao et al., 2017). Through the utilization of a microfluidic chip for analysis, scientists have observed three distinct patterns of TNT formation (Xiao et al., 2017). Furthermore, they have discovered that during cell migration, VACV-induced TNTs have the ability to elongate, facilitating faster cell-to-cell transmission (Xiao et al., 2017). Taken together, the loss of F11L expression and further exploration of viral components involved in TNTs may offer a potential strategy to counteract the spread of VACV through TNTs.

Ebola virus (EBOV), a negative-strand RNA virus, belongs to the enveloped filovirus family. Unlike Lassa virus (LASV), EBOV and other filoviruses are known to extensively utilize TNTs during their replication cycle. Through the use of fluorescent phalloidin treatment and antibodies, F-actin, M-Sec, and NP have been identified to enhance EBOV genome replication and transcription (Djurkovic et al., 2023). During infection, EBOV proteins such as VP40 and GP, along with the viral genome, localize to TNTs containing F-actin and tubulin. Intriguingly, EBOV nucleocapsids can induce the formation of TNTs and travel through them even in the absence of viral infection. Furthermore, EBOV demonstrates the ability to replicate despite blocking viral entry, mirroring other viruses that exploit TNTs for transmission (Djurkovic et al., 2023). However, it is still unclear how EBOV exploits TNTs and what factors are involved in their formation. A better understanding of these mechanisms could stimulate the development of antiviral methods for filoviruses.

## 3 Conclusion and perspectives

Cell-to-cell communication, a crucial process for maintaining tissue homeostasis and facilitating pathogen transmission, has been a subject of immense interest in the scientific community. In 2004, TNTs emerged as a novel mediator of intercellular transmission, triggering extensive discussions among researchers. However, the fragile nature of TNTs makes them susceptible to structural disruption during experiments. A recent study proposed the use of a microfluidic chip with side chambers to monitor nanotube formation and protect them from fluidic shear stress. Additionally, a new method employing protein-activated and FRET-enhanced excited-state intermolecular proton transfer fluorescent probes has been suggested for observing tunneling nanotubes in live cells (Zhu et al., 2023).

TNTs are typically composed of thin actin-rich structures, although some thicker ones also contain actin and microtubules. For instance, tunneling nanotubes induced by alphaherpesviruses consist of F-actin and tubulin, exhibiting stable characteristics that differentiate them from common nanotubes. TNTs exhibit slight differences when compared to other actin-rich structures as they are often not attached to the substrate. Similar cellular structures known as plasmodesmata have been observed in plants. Phalloidin is widely accepted as a marker for F-actin, binding tightly to and stabilizing actin, while alpha-tubulin is utilized for labeling tubulin. Further investigation is necessary to develop more specific markers for TNTs to distinguish them from other actin-rich structures.

It is widely recognized that TNTs play a crucial role in facilitating the rapid cell-to-cell transmission of viruses. These structures transport various viral components, including virions, viral genomic RNA, replicase proteins, and other viral proteins, to uninfected cells. Remarkably, viral components have been detected in the presence of virus-neutralizing antibodies, suggesting that TNTs provide an efficient spreading route for viruses to bypass certain steps and evade surveillance by the host immune system. However, it remains unclear whether these structures are open or closed-ended during viral infection. This distinction may depend on cell type-specific factors; for example, HIV dissemination occurs via closed TNTs that connect T cells, while TNTs between PC12 cells are open-ended. Limited information is available regarding whether this difference is virus-dependent. Furthermore, confirming the bidirectional or unidirectional transfer of viral-associated particles through TNTs is not always straightforward. For instance, GFP-tagged NS1 of IAV demonstrates unidirectional saltatory movement via TNTs, while Rab11a can mediate the bidirectional transport of vRNP within TNTs, facilitating cellular coinfection and reassortment.

The formation of TNTs is stimulated by various stressful conditions, including ROS, inflammation, and temperature, which can also influence the diameter of nanotubes depending on the cell types involved. However, further studies are needed to understand how cellular stresses modulate membrane structures through intracellular signaling pathways. The duration of intercellular contact also plays a role in TNT formation. Several viral proteins, such as HIV’s Nef, HTLV-1’s p8, and alphaherpesviruses’ US3, have been implicated in promoting TNT formation (Table 1). Additionally, multiple molecules are involved in the formation of TNTs during viral infection, as summarized in Table 1. The Arp 2/3 complex and other factors have been identified as central components in TNT formation. A recent study has linked Gi/o-coupled G-protein coupled receptors (GPCRs) to the development of TNTs in response to bioactive signaling lipids (Cooke et al., 2023). This study further demonstrated that the GPCR effector PI3K-regulated guanine nucleotide exchange factor FARP1 acts through CDC42 to promote TNT formation, revealing a novel signaling pathway for virus-induced TNT formation (Cooke et al., 2023). Inhibiting these factors can significantly reduce TNT formation and effectively decrease the spread of viruses. However, the precise mechanisms underlying TNT formation during viral infection, as well as the interaction between viral proteins and cytoskeleton proteins, are still not fully understood. Further advancements in sensitive imaging techniques are required to explore the internal structure of TNTs, and future studies are needed to elucidate the detailed intercellular nanotube transport process during viral infections, aiming to gain a comprehensive understanding of viral spread and pathogenesis.

Additionally, while many experiments have been conducted to study virus intercellular transmission *in vitro*, there is a lack of research exploring the transmission of viruses through TNTs *in vivo*. Of interest is the newly discovered SARS-CoV-2 virus, which utilizes TNTs as a route of spread, warranting investigation into whether these molecules play a significant role in TNT formation. Aside from TNTs, exosomes also serve as a mechanism for long-range communication. Although prior research has suggested that exogenous exosomes may induce TNT formation in tumor conditions (Thayanithy et al., 2014), their specific role in TNT occurrence during viral infection remains unclear.

TNTs are considered a direct and rapid pathway for viral infection, as well as for neurodegenerative and other pathological diseases. Therefore, it is crucial to develop strategies to interfere with this process. Various approaches have been explored to inhibit virus propagation through TNTs. For example, disrupting F-actin using cytochalasin D, inhibiting PAK1 with IPA-3, and neutralizing the low pH of endosomes with Bafilomycin A1 have all shown promise in reducing virus transmission via TNTs, making them potential candidates for antiviral strategies. Seeking drugs that can impede proteins required for TNT formation and target the viral genome replication step also holds potential as an effective approach. Additionally, lowering ATP levels to hinder the viral replication cycle and disrupt the transport of viral materials could open up new avenues for antiviral development. However, it is important to conduct further studies to mitigate the impact of F-actin-disrupting drugs on normal cells. Furthermore, most TNT blockers have not been tested in animal models, so it is crucial to investigate their efficacy *in vivo* and conduct clinical trials to evaluate their role in combating virus dissemination. In conclusion, while most studies have focused on exploring the role of TNTs in virus infection, it is equally important to delve into the specific processes involved in virus-mediated TNT formation, its effects on recipient cells, and understand the corresponding human immune responses. Such research endeavors may provide valuable insights for the development of novel antiviral drugs and the treatment of cancer and neurological disorders.

## Author contributions

WL: Writing – original draft. ZL: Writing – review & editing. SW: Writing – review & editing. JH: Writing – review & editing. LZ: Conceptualization, Funding acquisition, Writing – review & editing.
